# Arsenic Nanoparticles Trigger Apoptosis via *Anoikis* Induction in OECM-1 Cells

**DOI:** 10.3390/ijms25126723

**Published:** 2024-06-18

**Authors:** Alejandra A. Covarrubias, Mauricio Reyna-Jeldes, Seidy Pedroso-Santana, Sabrina Marín, Carolina Madero-Mendoza, Cecilia Demergasso, Claudio Coddou

**Affiliations:** 1 Departamento de Ciencias Biomédicas, Facultad de Medicina, Universidad Católica del Norte, Coquimbo 1781421, Chile; alejandra.covarrubias@ucn.cl (A.A.C.); mauricio.reynajeldes@oncology.ox.ac.uk (M.R.-J.); 2 Núcleo para el Estudio del Cáncer a Nivel Básico, Aplicado y Clínico, Universidad Católica del Norte, Coquimbo 1781421, Chile; seidy.pedroso@ucn.cl (S.P.-S.); smarin@ucn.cl (S.M.); cdemerga@ucn.cl (C.D.); 3 Millennium Nucleus for the Study of Pain (MiNuSPain), Santiago 8331150, Chile; 4 Facultad de Ciencias Agropecuarias, Universidad del Alba, La Serena 1700000, Chile; 5 Laboratory of Cancer Biology, Department of Oncology, Old Road Campus Research Building, University of Oxford, Oxford OX3 7DQ, UK; 6 Centro de Biotecnología “Profesor Alberto Ruiz”, Universidad Católica del Norte, Antofagasta 1200000, Chile; 7 Carrera de Medicina, Facultad de Medicina y Odontología, Universidad de Antofagasta, Antofagasta 1200000, Chile; caromadero16@gmail.com

**Keywords:** arsenic nanoparticles, *anoikis*, apoptosis, OECM-1, Bit-1, squamous cell carcinoma

## Abstract

Arsenic compounds have been used as therapeutic alternatives for several diseases including cancer. In the following work, we obtained arsenic nanoparticles (AsNPs) produced by an anaerobic bacterium from the *Salar de Ascotán*, in northern Chile, and evaluated their effects on the human oral squamous carcinoma cell line OECM-1. Resazurin reduction assays were carried out on these cells using 1–100 µM of AsNPs, finding a concentration-dependent reduction in cell viability that was not observed for the non-tumoral gastric mucosa-derived cell line GES-1. To establish if these effects were associated with apoptosis induction, markers like Bcl2, Bax, and cleaved caspase 3 were analyzed via Western blot, executor caspases 3/7 via luminometry, and DNA fragmentation was analyzed by TUNEL assay, using 100 µM cisplatin as a positive control. OECM-1 cells treated with AsNPs showed an induction of both extrinsic and intrinsic apoptotic pathways, which can be explained by a significant decrease in P-Akt/Akt and P-ERK/ERK relative protein ratios, and an increase in both PTEN and p53 mRNA levels and Bit-1 relative protein levels. These results suggest a prospective mechanism of action for AsNPs that involves a potential interaction with extracellular matrix (ECM) components that reduces cell attachment and subsequently triggers *anoikis*, an anchorage-dependent type of apoptosis.

## 1. Introduction

Cancer is a major global health concern that, despite enormous scientific and clinical efforts, has not shown significant decreases in its mortality rates over the years [1]. This permanent need for improvement has led to consider different therapeutic approaches [2,3]. One of these strategies is the association of standard chemotherapeutic drugs with nanoparticulated agents to subvert cancer drug resistance mechanisms and increase their overall selectivity against cancer cells, improving the safety concerns normally associated with chemotherapy. To succeed on these fields, nanotechnology allows the synthesis of novel therapeutic materials with particle sizes comparable to intracellular molecules (particle size < 100 nm) [4,5]. Due to these considerations and exceptional properties like mass density [6] and surface charge [7], nanoparticles have a relatively large surface area that enables their interaction and functionalization with various biomolecules, like DNA [8], RNA [9], peptides [10], aptamers [11], and antibodies [12], which help to exert their effect against cancer cells.

Arsenic compounds, such as arsenic trioxide (As_2_O_3_) and realgar (As_4_S_4_), have been used as therapeutic agents for more than 2000 years [13] to treat diseases like asthma, chorea, eczema, pemphigus, pernicious anemia, psoriasis, and Hodgkin’s lymphoma. Despite that radiotherapy and cytotoxic chemotherapy replaced the use of arsenic-based compounds as tools for cancer management in the early 20th century mainly due to safety concerns [14], As_2_O_3_ is still used in clinical set-ups, being a first-line agent in acute promyelocytic leukemia (APL) treatment [15,16,17]. Arsenic compounds are capable of inducing apoptosis of APL cells by altering reactive oxygen species balance and cell death-associated genes expression [13]. Because of their improved safety profiles, formulations of arsenic nanoparticles (AsNPs) have been proposed as potential cancer treatments [18]. AsNPs have been tested in a wide variety of cancer-derived cell lines such as breast (MCF-7 and MDA-MB-231) [19], APL (NB4) [20], melanoma (BOWES and A375) [21], and hepatocellular carcinoma (Huh7 and Bel-7402) [22], showing higher efficacy profiles than their counterparts with higher particle size. Realgar, a compound with better safety profile than As_2_O_3_, has also been tested in leukemias and solid tumors, and several nanoparticulated variants have been proposed to improve As_2_O_3_ solubility [23].

In nature, biogenic synthesis of metallic, non-metallic, and metalloid nanoparticles (NPs) such as Au, Ag, Zn, Se, Pt, Pd, Fe, Cu, Ni, Ti, and As nanoparticles can be achieved using plants [24] and microorganisms such as bacteria [25], algae [26], and fungi [27]. Biogenic NPs exhibit some advantages when compared to physicochemically synthesized NPs, like improved antimicrobial, anticancer, and larvicidal performances, among other applications [24]. This improvement in their efficacy is achieved by coating NPs with biological molecules, which makes them more biocompatible when compared with chemically produced NPs [26]. Moreover, biogenic NP production is a cost-effective and environmentally friendly alternative to synthesis. Biogenic AsNPs have been manufactured using microorganisms from the *Salar de Ascotán* [28], which are capable of reducing arsenate via arsenate respiration [29].

Several studies have shown that the antiproliferative effects of arsenic on cancer cells are caused by a combination of several factors, such as modulation of intratumoral mitochondrial integrity [22,30] and inflammatory response [31], reactive oxygen species generation [32], and the induction of pro-apoptotic genes and suppression of cell death resistance mechanisms [33]. Regarding skin lesions treatment, there is evidence both in vitro and in vivo that supports the use of arsenic compounds in psoriasis [34], plantar warts [35], and melanoma [36]. These findings portray AsNPs as a promising agent to be used as adjuvant therapy against other types of tumors where standard chemotherapy has limited success rates. Among this type of tumors is oral squamous cell carcinoma (OSCC), a malignancy developed at the oral and oropharyngeal cavity that has several etiological factors, smoking and alcohol consumption being among the most common, especially in the Western world, where its prevalence is around 1–4% of all cancers [37]. OSCC treatment alternatives include surgical excision/resection, radiotherapy, systemic cytotoxic chemotherapy, and the use of targeted agents like epidermal growth factor receptor (EGFR) inhibitors. However, despite these approaches, an important margin of 20–30% of surgically resected OSCC cases, even with tumor-free margins wider than 5 mm, have the chance to develop local or regional relapse. For this reason, OSCC has a 5-year overall survival (5-y OS) rate around 50% regardless of gender [38].

The following study describes for the first time the antitumoral effects of biogenic AsNPs [39] using OECM-1 cells as a preliminary in vitro model for OSCC and explains the cellular mechanisms behind their activity. AsNPs showed a cytotoxic effect on OECM-1 cells, and these effects can be explained by the induction of *anoikis*, a programmed cell death mechanism triggered by cell detachment from the extracellular matrix (ECM).

## 2. Results

### 2.1. AsNP Preparation and Characterization

**Figure 1:**
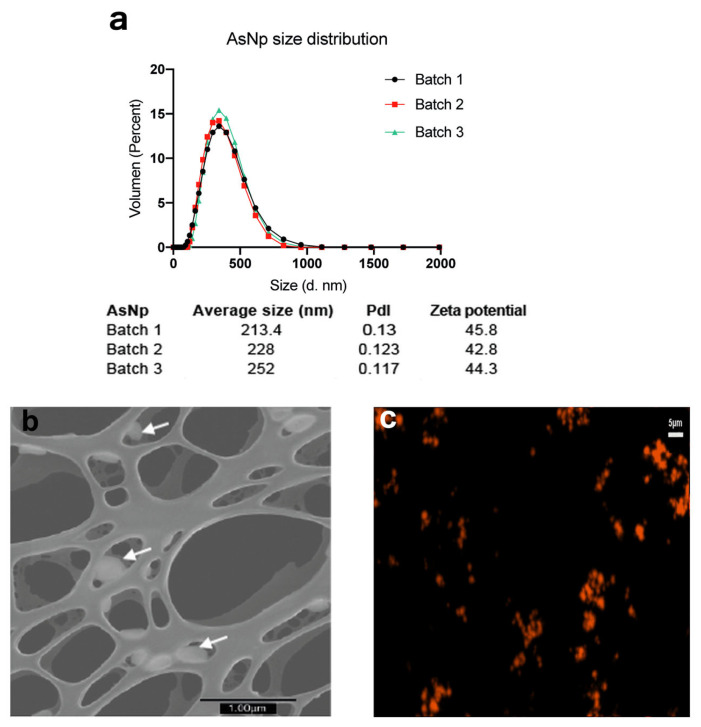
AsNP characterization. (**a**) DLS and Zeta potential analysis for three independent AsNP batches (PdI: polydispersity index). (**b**) S-TEM micrograph of AsNPs. White arrows indicate AsNPs. (**c**) Fluorescent microscopy image of agglomerated AsNPs effectively conjugated with TRITC.

Three independent AsNP batches were produced and characterized (Figure 1).

Overall, average particle size was ≈200 nm, with a positive zeta potential of approximately 40–45 mV, a polydispersity index (pdl) < 0.15 (Figure 1a), and a final arsenic concentration between 200 and 300 ppm. The latter values were used to calculate AsNP concentrations for the subsequent in vitro experiments. The production of biogenic realgar AsNPs involves a low batch-to-batch variability (Figure 1a) [39]. Under the microscope, AsNPs showed a quasi-spherical appearance (Figure 1b), and their conjugation with TRITC generated bright orange fluorescent nanoparticles (Figure 1c). These biogenic AsNPs showed a storage stability at 4 °C higher than 100 days. Variations in size, pdl, and zeta potential observed for one batch of AsNPs between days 1 and 107 after synthesis were 188.5, 0.126, 47.2, and 108.5, 0.134, and 47.9, respectively.

### 2.2. AsNPs Inhibit OECM-1 Cell Viability by Compromising Cell Adhesion

**Figure 2:**
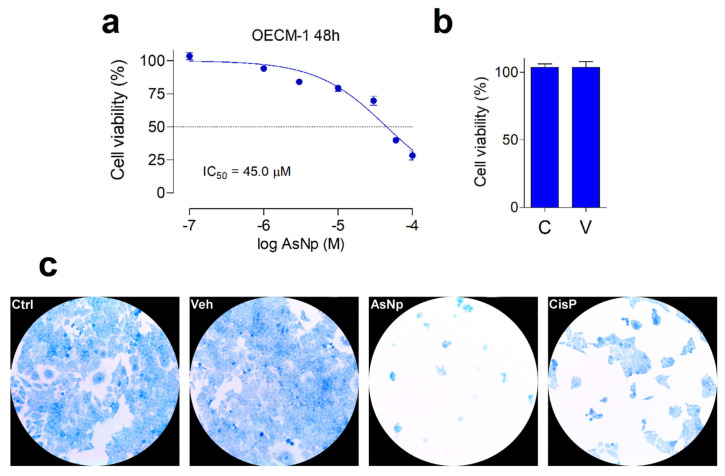
AsNP effects on OECM-1 cell viability. (**a**) Changes in cell viability, measured by resazurin reduction assay, obtained for 48 h treatments with 1–100 µM of AsNPs. (**b**) Cell viability in control cells (C) and in vehicle-treated cells (V), 0.03% chitosan for 48 h. (**c**) Loss of adherence of OECM-1 cells incubated with vehicle, 60 µM of AsNPs and 100 µM of CisP for 48 h, and stained with 0.2% methylene blue solution. In (**a**,**b**), data are expressed as mean ± SEM of 6 independent experiments.

The cytotoxic effect of AsNPs against OECM-1 cells was established by resazurin reduction assay. The cell viability and cell morphology were recorded using a 1–100 μM AsNP concentration range and 48 h incubations (Figure 2).

In general, a concentration-dependent decrease in cell viability was observed for AsNPs. Maximal proliferation inhibition was 75% for 100 µM of AsNPs, estimating an IC_50_ = 45.1 ± 3.0 μM (Figure 2a), these effects being totally absent when OECM-1 cells were incubated with the AsNP solubilization vehicle 0.03% chitosan (Figure 2b). To establish if the AsNP effects are selective against cancer cells, GES-1 cells were included as a non-tumoral control. On these cells, 100 μM of AsNPs, the highest concentration tested, only caused a 35% inhibition in viability, which translates into an IC_50_ > 100 μM (Supplementary Figure S1). In addition to these observations, AsNP-treated OECM-1 cells exhibited a noticeable detachment that reduced cell counts after 48 h treatments. These preliminary observations on cell adhesion were confirmed by methylene blue staining, which confirmed that, after AsNP treatment, OECM-1 cells detached from their dishes and became round-shaped, losing their original polygonal morphology (Figure 2c and Supplementary Figure S3). Conversely, no morphological changes were observed when OECM-1 cells were treated with cisplatin (CisP; Figure 2c). To quantify these observations, we determined the percentage of cells in three different sections of the culture obtaining the following results: 81.0 ± 6.4% in control cells (Ctrl); 89.0 ± 4.6% in vehicle-treated cells (Veh); 3.0 ± 1.6% in AsNP-treated cells (AsNPs); and 31.0 ± 3.2% in cisplatin-treated cells (CisP).

### 2.3. AsNPs Induce Apoptosis in OECM-1 Cells

**Figure 3:**
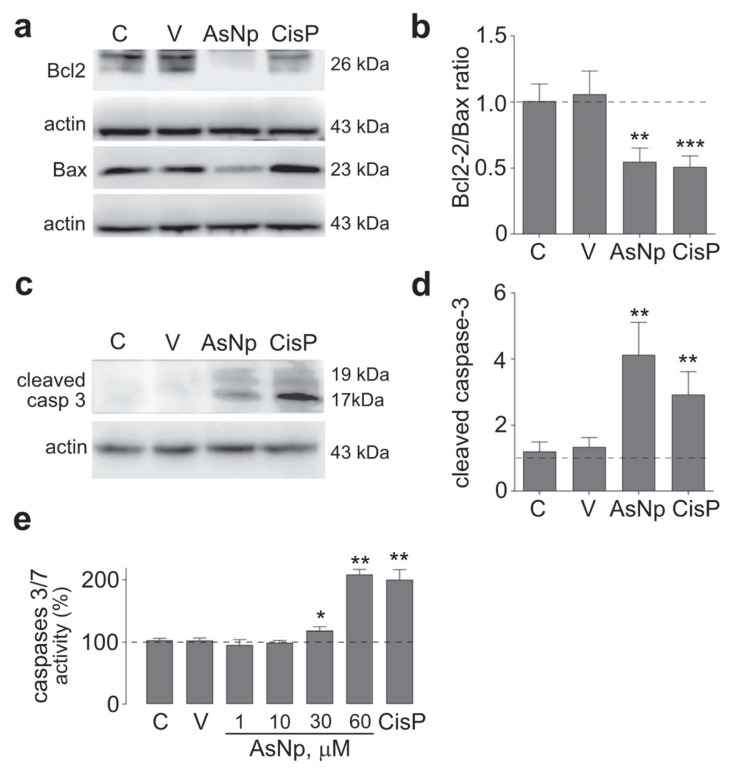
AsNP effects on apoptosis induction. (**a**) Bcl-2 and Bax immunodetection in OECM-1 cells incubated with 0.03% chitosan (vehicle), 60 µM of AsNPs, and 100 µM of CisP for 48 h. (**b**) Relative Bcl2/Bax protein ratio quantification. (**c**) Cleaved caspase 3 immunodetection in OECM-1 cells incubated with vehicle, 60 µM of AsNPs, and 100 µM of CisP for 48 h. (**d**) Densitometric analysis of figure (**c**). (**e**) Caspase 3/7 activities assay in OECM-1 cells incubated with 1, 30, 60, and 100 µM of AsNPs for 48 h. Data are expressed as mean ± SEM of 4 independent experiments. Parametric analysis was performed by Student’s *t*-test. Significance level was established at *p* < 0.05 (*), 0.001 ≤ *p* < 0.01 (**) and *p* < 0.001 (***).

After these initial experiments, the possibility that AsNPs reduced cell proliferation through apoptosis induction on OECM-1 cells was explored. With this aim, protein levels of several apoptotic markers were evaluated by treating cells with 60 μM of AsNPs (concentration capable of reducing cell viability by ≈50%), 0.03% chitosan vehicle, and 100 μM of cisplatin (as a positive control) for 48 h. Initially, intrinsic apoptotic pathway mediators were assessed by establishing a protein level ratio between Bcl-2 (as anti-apoptotic marker) and Bax (as pro-apoptotic marker), observing a significant decrease in this ratio for both AsNP and cisplatin treatments (*p* < 0.01 and *p* < 0.001, respectively; Figure 3a,b). In addition, cleaved caspase 3 relative protein levels were determined as an active executor caspase marker, finding that both AsNP and cisplatin treatments significantly increased cleaved caspase 3 protein levels (*p* < 0.01 and *p* < 0.001, respectively; Figure 3c,d).

**Figure 4:**
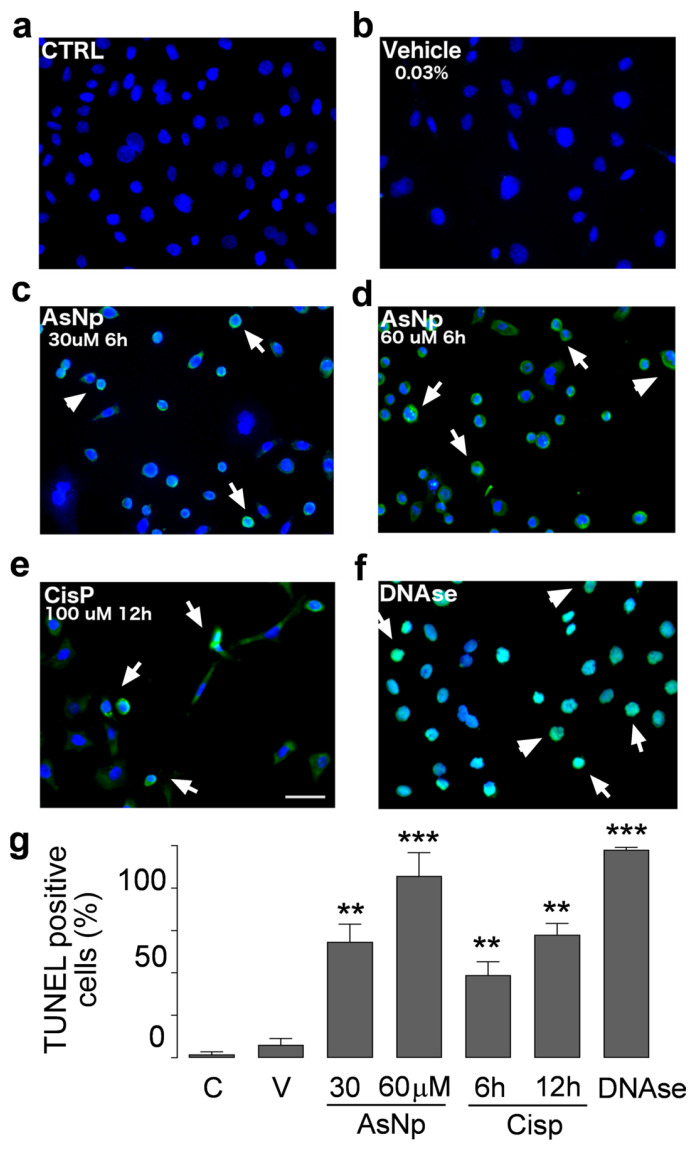
AsNP activity on apoptotic DNA fragmentation. TUNEL assay in untreated control (**a**), 0.03% chitosan (vehicle, (**b**)), and 30 and 60 µM AsNP treatments for 6 h ((**c**,**d**), respectively) was performed in OECM-1 cells. Positive controls correspond to 100 µM CisP for 12 h (**e**) and DNase I for 6 h (**f**). White arrows in (**c**–**f**) represent TUNEL-positive cells examples for each treatment condition. (**g**) Percentages of TUNEL-positive cells obtained by quantifying 100 cells per condition. C = control; V = vehicle). Parametric analysis was performed via Student’s *t*-test. Significance level was established at 0.001 ≤ *p* < 0.01 (**) and *p* < 0.001 (***).

To support the findings observed for cleaved caspase 3, executor caspases 3/7 activity was determined under different concentrations of AsNPs, observing a significant increase from 30 μM of AsNPs onwards (*p* < 0.05, Figure 3e). Lastly, late-stage apoptotic events were evaluated in OECM-1 cells via TUNEL assay (Figure 4).

In control and vehicle conditions (Figure 4a,b), we did not observe DNA fragmentation; in contrast to that observed with 30 and 60 μM AsNP treatments (Figure 4c,d). For these experiments, AsNP incubation time was reduced to 6 h to obtain a greater number of cells adhered to coverslips. Positive controls using CisP and DNAase also induced DNA fragmentation (Figure 4e,f). Considering this, a significant increase in TUNEL-positive cell percentage was observed after both 30 and 60 μM AsNP treatments (54.7 ± 16.6% and 85.8 ± 21.9%, respectively; *p* < 0.01, Figure 4g). When comparing the TUNEL-positive cells’ percentages obtained for the vehicle and medium-treated conditions, no significant differences were found (Figure 4a,b). Lastly, cisplatin showed 39.0 ± 12.5% and 58.1 ± 10.6% of TUNEL-positive cells after a 6 and 12 h treatment, respectively (Figure 4g).

### 2.4. AsNPs Induce Anoikis Signaling in OECM-1 Cells

**Figure 5:**
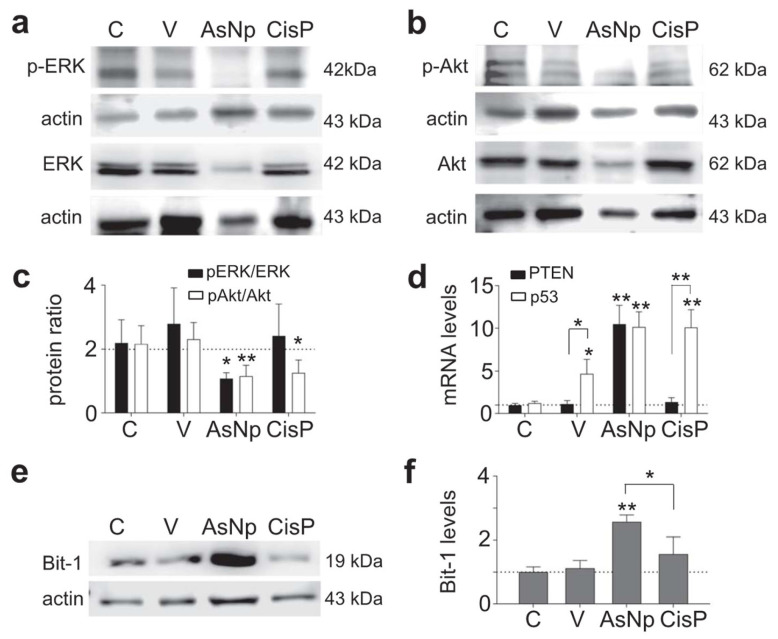
AsNPs trigger apoptosis via *anoikis*. (**a**,**b**) Erk, p-Erk, Akt, and p-Akt immunodetection in OECM-1 cells incubated with 0.03% chitosan (vehicle), 60 µM of AsNPs, and 100 µM of CisP for 48 h. (**c**) Densitometric analysis of p-Akt/Akt and p-Erk/Erk relative protein ratios. (**d**) Quantification of PTEN and p53 relative expression levels in OECM-1 cells using qPCR. (**e**) Bit-1 immunodetection in OECM-1 cells incubated under the same experimental conditions. (**f**) Densitometric analysis of figure (**e**). Data are expressed as mean ± SEM of 4 independent experiments. Parametric analysis was performed by Student’s *t*-test. Significance level was established at *p* < 0.05 (*) and 0.001 ≤ *p* < 0.01 (**).

To establish if the pro-apoptotic signaling triggered by AsNP treatment on OECM-1 cells is linked to their detachment from the ECM, additional assays involving specific features of anoikis signaling were performed in Western blot (Figure 5).

As it is widely known, *anoikis* modifies classic cell survival pathways like Erk and Akt, thus their relative phosphorylation ratios were evaluated via Western blot, finding that the p-Erk/Erk protein ratio decreased significantly in cells incubated with AsNPs and cisplatin (*p* < 0.01 and *p* < 0.05, respectively; Figure 5a,c). When the p-Akt/Akt protein ratio was studied, a decrease in its phosphorylated form was observed only in the AsNP-treated condition (*p* < 0.05, Figure 5b,c). In addition, there were no significant differences at both ratios when the vehicle-treated and untreated conditions were compared (*p* = 0.299 to pAkt/Akt, and *p* = 0.650 to pErk/Erk, Figure 5c). This Akt phosphorylation reduction observed in AsNP-treated OECM-1 cells was consistent with the increase in PTEN expression observed only for AsNP treatments (Figure 5d). Regarding p53 expression levels, a significant increase in its mRNA levels was observed for the 0.03% chitosan- (*p* < 0.05), AsNP- (*p* < 0.01), and CisP-treated conditions (*p* < 0.01; Figure 5d). To evaluate an *anoikis*-specific protein marker, Bit-1 relative protein levels were established via Western blot, observing a significant increase in Bit-1 relative levels in the presence of AsNPs (*p* < 0.001; Figure 5f), this effect being absent after CisP treatment (*p* = 0.453; Figure 5e,f).

### 2.5. Interactions of AsNPs with OECM-1 Spheroids

**Figure 6:**
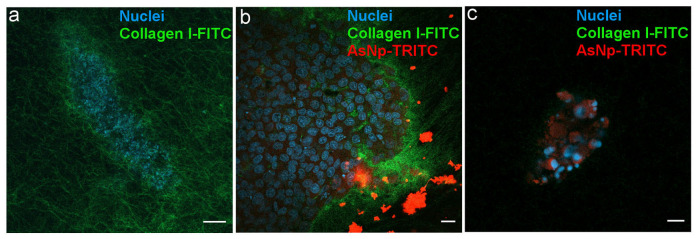
AsNPs interact with the ECM in OECM-1 cell spheroids. (**a**) Spheroids were created using a matrix containing 90% type I collagen and 10% FITC-type I collagen. (**b**) Spheroids incubated with 60 µM of TRITC-conjugated AsNPs for 1 h. (**c**) Small spheroids under similar treatment conditions. Magnification bars: (**a**,**c**) = 10 µm; (**b**) = 20 µm.

To further explain the mechanisms behind AsNP pro-apoptotic effects, it is relevant to establish if these nanoparticles exert their effects by interacting with OECM-1 cells at intra- and/or extracellular levels. For this, spheroids cultured in a collagen matrix with 10% FITC-conjugated type I collagen were incubated with 60 μM of TRITC-conjugated AsNPs for 30 min. The confocal image shows the interaction of AsNPs at the 3D model of the OECM-1 cells sphere (Figure 6).

A homogeneously distributed fluorescent collagen matrix was observed around the 3D OECM-1 culture in the untreated condition (Figure 6a). In AsNP-treated cells, an important disruption of these collagen matrix assemblies, noticed by the evident loss of FITC signal, was observed (Figure 6c). When the distribution of the TRITC signal was analyzed, both intra- and extracellular staining was found, finding that AsNP-TRITC aggregates outside large OECM-1 spheroids (Figure 6b) and shows a greater intracellular TRITC intensity in smaller spheroids, where the nuclei of the cells exhibit an apparent degree of fragmentation (Figure 6c). Finally, and in order to discard if TRITC conjugation can compromise AsNP antiproliferative activity on OECM-1 cells, additional resazurin reduction assays were performed, finding that TRITC-conjugated AsNPs have similar effects as their non-conjugated counterpart (Supplementary Figure S2).

## 3. Discussion

With more than 10 million annual deaths, cancer is an ongoing global concern that increases as life expectancy increases worldwide. For this reason, novel therapeutic and/or adjuvant strategies are critical to controlling this disease [40]. One promising strategy is the development of specific, safe, and efficient nanoparticles for cancer treatment [2]. NPs can be used as a carrier for known or novel anticancer agents, or they can interact directly with cancer cells, triggering cell death as reported for mineral nanoparticles [3,4,5]. For example, arsenic trioxide (As_2_O_3_) and realgar arsenic nanoparticles are capable of inducing cytotoxic effects on various cancer cell models [5,19,20,21]. The polymer-coated AsNPs used in this work showed an average size between 200 and 250 nm (Figure 1), determined by DLS, which has been described as a proper size for cell internalization [41,42,43]. We can see this in our results, where the TRITC-conjugated AsNP signal can be found at both intra- and extracellular levels (Figure 6b,c). Extracellular agglomerates of TRITC-AsNP signal could be explained by the interaction of the cationic polymer layer of AsNPs with the negatively charged cell membrane.

Our results showed antiproliferative effects for the human OSCC cell line OECM-1, in which biogenic AsNPs inhibited cell proliferation with an IC_50_ ≈ 45 μM after a 48 h treatment. This is the first report about the effect of AsNPs (biogenic or non-biogenic) in OSCC cell lines. This effect is similar but more potent than the activities reported for a 72 h treatment of non-biogenic AsNPs in different cancer-derived cell lines such as MCF-7 (breast cancer), HepG2 (hepatocellular carcinoma), and A549 (lung carcinoma) cells (IC_50_ = 39.9, 34.3, and 30.3 μM, respectively) [5]. Biogenic AsNP potency is even higher than the cytotoxicity levels observed for the OSCC cells CAL 27, HSC 3, and SCC 4 treated with arsenic trioxide (ATO) for 72 h (IC_50_ > 126.4, 37.4, and >126.4 μM, respectively) [44]. In contrast, biogenic AsNPs only partially inhibited cell proliferation of the non-tumoral gastric mucosa cell line GES-1 (IC_50_ > 100 μM), which supports the notion that these AsNPs have a selective antiproliferative effect against cancer-derived cells. These observed differences could be due to the enhanced capacity of cancer cells to incorporate nanoparticles when compared to control cells [45]. In the study by Azizi and colleagues, LD_50_ of albumin-coated silver NPs (AgNPs) was found to be several times lower for human breast cancer cell lines (MDA-MB-231 and MCF-7) than the human non-tumoral breast epithelia cell line MCF10A and primary human white blood cell cultures [46]. These authors found that a greater amount of AgNPs could be found inside the studied breast cancer cell lines, which led to an increase in reactive oxygen species that induced mitochondrial damage and subsequent apoptosis [45]. In a similar fashion, our experiments revealed that OECM-1 cells are capable of incorporating AsNPs, as shown in Figure 6.

The process by which these nanoparticles induce apoptosis could involve different mechanisms, since we observed increased activity of both executor caspases 3 and 7 in these cells, a contribution of both extrinsic and intrinsic pathways. Several mechanisms have been proposed to explain the pro-apoptotic action of nanoparticles in cancer cells, such as reactive oxygen species increase (ROS), up- and down-regulation of proteins, immunological interventions, inhibition of transcription, site-specific cytotoxicity, among others [47,48]. Both arsenic compounds and arsenic nanoparticles can increase ROS, leading to a decrease in the mitochondrial membrane potential, resulting in an increased expression of Bax and subsequent cytochrome c release and subsequent apoptosis induction via intrinsic pathway [47,48]. In OECM-1 cells, we confirmed the involvement of the intrinsic pathway by the decrease in the Bcl2/Bax ratio in cells exposed to AsNPs (Figure 3). Although we did not quantify ROS levels in our study, this Bcl2/Bax imbalance is capable of inducing apoptosis. This pro-apoptotic activity was further confirmed by TUNEL assay, which demonstrated that 30 and 60 μM of AsNPs also induced DNA fragmentation (Figure 4).

Next, we studied in more detail the specific type of apoptosis induced by AsNPs. Because our primary findings showed that AsNPs not only killed but also quickly detached OECM-1 cells (Figure 2), we tested if AsNPs induced apoptosis via inducing *anoikis*, a particular type of apoptosis that is triggered by loss of cell anchorage to the ECM. To prove this, first we measured changes in protein phosphorylation of Erk and Akt kinases, which mediate cell processes related to proliferation, promoting cell survival when activated, and whose function is affected during *anoikis* [49,50]. Consistent with these reports, we observed that AsNPs decreased both Erk and Akt phosphorylation, and also decreased the relative levels of these proteins (Figure 5). It has been demonstrated in other studies that arsenic compounds can induce changes at protein kinase levels [21,51]. Pastorek and collaborators observed that the modulation of Akt, Erk1/2, and IκB kinases were differentially induced in the BOWES melanoma cell line, depending on the type of arsenic compound involved. These signaling pathways are central to various cellular responses [21]. The Erk1/2 pathway is capable of inhibiting apoptosis in response to a wide variety of stimuli, such as tumor necrosis factor (TNF), Fas ligand, TNF-related apoptosis-inducing ligand (TRAIL), radiation, osmotic stress, hypoxia, among others [52]. Similarly, the PI3K/Akt pathway is one of the most potent intracellular mechanisms to promote cell survival [53]. For example, in a study that analyzed four downstream effectors of growth factor receptors, PI3K, Ras, Raf, and Src, PI3K was the only one capable of inhibiting apoptosis after serum withdrawal [53,54].

To further confirm that AsNPs induce *anoikis* in OECM-1 cells, we measured the expression levels of phosphatase and tensin homolog (PTEN). PTEN is a phosphatase that acts as a tumor suppressor gene that negatively regulates the Akt signaling pathway [53,54]. We found a robust increase in the expression of PTEN in AsNP-treated OECM-1 cells, which is in agreement with its described antitumor activity. Finally, we measured a specific *anoikis* marker, Bit-1, a 179-residue mitochondrial protein [55]. This protein is released from mitochondria when cells lose their anchorage and forms a complex with the transcriptional regulator protein Amino-terminal Enhancer of Split (AES). The formation of the Bit1-AES complex initiates a caspase-independent form of apoptosis [55,56]. The expression of this protein was augmented in AsNP-treated but not in CisP–treated cells, indicating that only AsNPs can induce *anoikis*. Interestingly, both AsNPs and CisP increased the expression of p53, a tumor suppressor gene, indicating that AsNPs can induce apoptosis by triggering both *anoikis*-specific and canonical pro-apoptotic pathways. In a final set of experiments, we observed the morphological changes induced by AsNPs on OECM-1 spheroids (Figure 6). Here, AsNP treatment disrupted the collagen matrix in which spheroids were embedded, further supporting the notion that AsNPs can have multiple effects, both promoting detachment via interaction with ECM and inducing cell death via *anoikis*, and enhancing other pro-apoptotic pathways via AsNP uptake. The former AsNP effects on *anoikis* induction can explain the results of previous reports showing that similar AsNPs prevent bone colonization by metastatic breast cancer by affecting their migration and invasion capabilities [57,58,59].

Regarding oral and skin cancer treatment, there is evidence of the potential advantages of using nanoparticles against these malignancies [60,61]. Teraoka and co-workers found that using 1.0 nM of gold nanoparticles combined with 4 Gy X-ray irradiation significantly reduced the total number of cells compared to 4 Gy X-ray irradiation alone in the human head and neck carcinoma cell line HSC-3. Furthermore, chitosan has been used as a drug delivery system for skin cancer treatment, being a versatile polymer with favorable properties [62,63,64], even with some reports showing its capability to induce p53 expression [65], which is something that was also found in our qPCR results for OECM-1 cells treated with 0.03% chitosan. Similarly, there is evidence of an antiproliferative effect and apoptosis induction of arsenate and As_2_O_3_, on A375 melanoma-derived cells [66]. Moreover, realgar nanoparticles are also able to induce apoptosis and autophagy in this cell line [66]. Future experiments should determine in preclinical and clinical models if these AsNPs could be useful as an alternative therapeutic approach for oral and skin cancer treatment.

## 4. Materials and Methods

### 4.1. Nanoparticles Preparation and Characterization

AsNPs were produced through the recently described metabolism of the anaerobic bacteria *Fusibacter ascotence* [29], isolated from the *Salar de Ascotán*. The AsNPs were then purified by acid treatment, centrifugation, and sonication, following the methodology described by Demergasso et al. [39] with some modification because Chitosan (0.2%) was used as the nanoparticles’ stabilizing agent. AsNPs were characterized by scanning transmission electron microscopy (S-TEM), dynamic light scattering (DLS), X-ray diffraction (DRX), and zeta potential measurements to analyze their shape, size, polydispersity index (pdl, stability on size distribution), mineralogy, and surface charge. For S-TEM studies, 0.1 g of each dried sample of nanoparticles were suspended in 1 mL of ethanol and deposited on the sample holder. The images were acquired using FSEM Hitachi SU5000 equipment (Hitachi, Japan) coupled to a Deben’s STEM detector. DLS and zeta potential analysis were performed on AsNP suspensions using a Zetasizer Nano ZS 90 instrument (Malvern Panalytical, Malvern, Worcestershire, UK). Briefly, concentrated AsNPs were diluted in the same buffer used for the polymer addition for DLS measurements. The total arsenic content in every AsNP batch was measured using a Millennium Excalibur spectrometer (PS Analytical, Orpington, Kent, UK). During manufacture, the treatment of nanoparticle surfaces with chitosan (0.2%) as a cationic agent provided amino groups [67] for subsequent functionalization. Tetramethylrhodamine-5-isothiocyanate (TRITC; Thermo Fisher Scientific, Waltham, MA, USA), an orange fluorescent agent, was linked to the amino groups as previously reported [68]. After conjugation, reaction was dialyzed to remove the excess of TRITC, and the obtained fluorescent AsNPs were stored at 4 °C.

### 4.2. Cell Culture

Human oral squamous carcinoma cell line OECM-1 was acquired from AddexBio (RRID:CVCL_6782; C0050019; AddexBio, San Diego, CA, USA), and human non-tumoral gastric epithelial cells (GES-1) were kindly donated by Dr. Dawit Kidane from the University of Texas at Austin, USA. Cells were maintained using Dulbecco’s Modified Eagle Medium (DMEM; Corning, Corning, NY, USA) supplemented with 10% fetal bovine serum (Biological Industries, Beit HaEmek, Israel), 1% of a 100,000 U/mL penicillin and 100,000 μg/mL streptomycin antibiotic solution (Corning, Corning, NY, USA). Cells were cultured in plastic cell culture dishes and flasks at 37 °C in a humidified atmosphere containing 5% CO_2_.

### 4.3. Resazurin Reduction Assay

OECM-1 cells were seeded in 96-well plates (Corning, Corning, NY, USA) following a 3000 cells/well proportion to achieve 40–50% confluence after an overnight incubation at 37 °C under a 5.0% CO_2_ atmosphere to promote cell adhesion. After this, cells were treated using an AsNP concentration gradient ranging from 1 to 100 μM for 48 h. In preliminary experiments, we tested 24 and 48 h incubation times, finding optimal effects with 48 h incubation. An additional untreated control consisting of a 0.03% chitosan (used as an AsNP suspension vehicle) in supplemented DMEM solution was included. After incubation, treatments were replaced with a 70 μM resazurin (Sigma-Aldrich, St. Louis, MO, USA) in supplemented DMEM solution. Fluorescence measurements were performed after a 4 h incubation at 37 °C under a 5.0% CO_2_ atmosphere. Relative Fluorescence Unit (RFU) measurements were performed using a NOVOstar Plate Reader (BMG Labtech, Ortenberg, Germany), detecting 570 nm and 590 nm as excitation and emission wavelengths, respectively. Cell proliferation percentages were obtained by comparing AsNP-treated RFU measurements with those acquired for the vehicle control. Median inhibitory concentrations (IC_50_) for AsNPs were calculated for every cell batch treated with 1–100 μM of AsNPs. Each functionality curve was adjusted to an exponential fit using GraphPad Prism (RRID:SCR_002798) version 9.0 (GraphPad Software, San Diego, CA, USA), and final IC_50_ values were obtained by calculating the mean ± S.E.M. of all the batches tested.

### 4.4. Methylene Blue Staining

OECM-1 cells were seeded (100,000 cells/dish) on 35 mm dishes in order to achieve 50–60% cell confluence after an overnight incubation at 37 °C under a 5.0% CO_2_ atmosphere. After this, dishes were treated for 48 h with 60 μM of AsNPs, 100 μM of cisplatin (CisP), which was used as a positive control, and 0.03% chitosan in supplemented DMEM as a vehicle control. In addition, a control group without any treatment was used to calculate the basal proliferation. After treatment, culture medium was discarded from each dish, and cells were washed with ice-cold PBS, fixed with 50% ice-cold ethanol for 10 min, and stained with a 0.2% methylene blue solution for 5 min. Afterwards, cells were washed twice with ice-cold PBS and visualized using a Leica optical microscope (DM500, Leica Microsystems, Wetzlar, Germany), acquiring images with an ICC50W camera (Leica Microsystems, Wetzlar, Germany). For cell quantification, three different 1091 × 944 pixel sections were chosen for each immunostaining after treatment, and the percentage of cells was established by calculating the total cell-covered area on each section using Image J v1.52a software (RRID:SCR_003070; National Institutes of Health, Bethesda, MD, USA).

### 4.5. Immunoblotting

OECM-1 cells were seeded on 100 mm dishes in order to reach 80–90% cell confluence after an overnight incubation. Cells were incubated for 48 h with 60 μM of AsNPs, 0.03% chitosan in supplemented DMEM (vehicle), and 100 μM of cisplatin as a positive control. Later, cells in dishes were homogenized using RIPA buffer, and total protein concentration was measured using the bicinchoninic acid assay with bovine serum albumin as the standard (Thermo Fisher Scientific, Waltham, MA, USA). Due to the loss of cell adhesion after AsNP treatment, culture medium with suspended cells was centrifuged at 900 g for 10 min, and the lysis buffer was added to the obtained pellet. Protein samples (40 or 50 μg/lane) were run on 10% SDS-polyacrylamide gels and transferred to polyvinylidene fluoride (PVDF) membranes (Thermo Fisher Scientific, Waltham, MA, USA) using a Mini Trans-Blot (Bio-Rad, Hercules, CA, USA) device. Blocking was performed using a 5% skimmed milk in TBS-Tween 20 (0.01%) solution for 30 min, and membranes were then incubated with anti-Bcl2, anti-Bax, anti-BID, anti-AKT (1/2/3), anti-Erk (1/2), anti-pErk (1/2), anti-Bit-1, and anti-Actin antibody solutions overnight at 4 °C. After primary antibody incubation, membranes were washed with TBS-Tween 20 solution and then incubated for 2 h at room temperature with HRP-conjugated secondary antibodies against rabbit or mouse IgG. In the particular case of anti-cleaved caspase 3 and pAKT (1/2/3) primary antibodies, 5% BSA in PBS-Tween 20 (0.01%) was used as a blocking solution, and antibody dilutions and membrane washes were performed using PBS-Tween 20. After secondary antibody incubation and washing, specific bands were revealed using a SuperSignal West Pico Plus Chemiluminescent Substrate (34580, Thermo Fisher Scientific, Waltham, MA, USA), and images were acquired using a C-Digit Blot Scanner documentation system (LI-COR Biosciences, Lincoln, NE, USA). Quantitative immunoblotting analysis was carried out using Image J (RRID:SCR_003070) v1.52a software (National Institutes of Health, Bethesda, MD, USA). Details about the antibodies used for these experiments are summarized in Supplementary Table S2.

### 4.6. Caspase 3/7 Activity Assay

Caspase 3/7 activity was quantified by seeding OECM-1 cells at a density of 5000 cells/well in flat-bottom white opaque 96-well plates (136101; Thermo Fisher, Waltham, MA, USA) and incubated overnight at 37 °C under a 5.0% CO_2_ atmosphere for cell attachment. The next day, cells were treated with a range of 1–100 μM of AsNPs for 48 h. As control conditions, caspase 3/7 activity was measured in cells treated with 0.03% chitosan in supplemented DMEM (vehicle control), 100 μM of cisplatin (positive control), and supplemented DMEM (negative control) solutions. After this incubation, culture medium was removed, and caspase 3/7 activity was measured using a Caspase-Glo^®^ 3/7 Assay kit (G8090; Promega, Madison, WI, USA), following the manufacturer’s instructions. In short, the activity of executor caspases in the samples can cleave the pro-luciferin-DEVD substrate provided in the kit, releasing aminoluciferin, which acts as substrate for luciferase, producing a chemiluminescent signal in the presence of ATP. Relative Light Unit (RLU) assessments were performed using a NOVOstar Plate Reader (BMG Labtech, Ortenberg, Germany), and relative caspase 3/7 activities were calculated as the RLU ratio between AsNP-treated, CisP-treated, or vehicle-treated conditions with their corresponding negative control condition.

### 4.7. TUNEL Assay

Cells were seeded on 12 mm coverslips and incubated with 30 and 60 μM of AsNPs for 6 h, using 0.03% chitosan in supplemented DMEM and 100 μM cisplatin solutions as vehicle and positive control conditions, respectively. Terminal deoxynucleotidyl transferase dUTP nick-end labeling (TUNEL) assay was carried out using the Click-iT TUNEL Alexa Fluor Imaging Assay kit (C10245; Thermo Fisher, Waltham, MA, USA) in accordance with the manufacturer’s instructions. Fluorescence images were captured using an Eclipse Ts2R-FL inverted fluorescence microscope equipped with a DS-Fi3 Camera (Nikon, Tokyo, Japan). Analysis was performed using Image J1.52a software (National Institutes of Health, Bethesda, MD, USA), transforming images to grayscale to quantify TUNEL-positive cells.

### 4.8. qPCR

To guarantee quality and reproducibility in these procedures, MIQE Guidelines [69] were applied to every step of the following methodology. OECM-1 cells were seeded on 60 mm culture dishes at a proportion of 50,000 cells/dish to achieve a 60–80% confluence after a 24 h incubation. After cell attachment, mRNA was extracted using TRIzol reagent (Invitrogen, Waltham, MA, USA) according to the manufacturer’s protocols. Yield and OD purity ratios (260 nm/280 nm and 260 nm/230 nm) were established using a NanoDrop One microvolume spectrophotometer (Thermo Fisher Scientific, Waltham, MA, USA). Reverse transcription was performed using an Affinity Script qPCR cDNA Synthesis kit (Agilent Technologies, Santa Clara, CA, USA), employing 1.0 μg of total RNA per reaction and including a no-retrotranscriptase control (−RT) for each sample. For qPCR reactions, Brilliant II SYBR^®^ Green QPCR Master Mix (Agilent Technologies, Santa Clara, CA, USA) was used, with 50 ng of cDNA and 300 nM as the final primer concentration for each one of the studied genes. PTEN and p53 were defined as target genes, and B2M was used as a referential gene. Primer design was performed using Primer-Blast, choosing primer pairs with an annealing temperature of 60 °C and capable of generating amplicon sizes of 50–250 bp. Primer sequences and characteristics are detailed in Supplementary Table S1. The thermal protocol used for all of these experiments consisted in an initial denaturation step of 95 °C for 10 min, followed by an amplification phase of 40 cycles of 30 s at 95 °C and 60 s at 60 °C, detecting fluorescence at the end of each cycle. After amplification, melting curves were performed on the amplified products, incubating them at 95 °C for 60 s, ramping down to 55 °C, and then increasing temperature to 95 °C at a rate of 0.2 °C/s, measuring fluorescence data continuously. To calculate relative gene expression, efficiency curves were performed for each gene, choosing the most proper calculation method between the equations proposed by Livak and Schimittgen [70] or Pfaffl [71] according to the differences in the amplification efficiency of each one of the analyzed genes.

### 4.9. 3D Culture of OECM-1 Cells Spheroids

3D culture of OECM-1 cells was performed partially following the hanging-drop method described by Chen et al. [72]. Cells were suspended in RPMI medium supplemented with 10% FBS, 1% penicillin–streptomycin solution and 2% Matrigel (354234; Corning, Corning, NY, USA). Then, 30 μL droplets containing ~3000 OECM-1 cells were added on the inner surface of the lid of 60 mm dishes. After this, the bottom part of each of these dishes was treated with 3 mL of PBS to prevent droplets from drying. With this configuration, lids were carefully placed over the bottom of each dish and incubated at cell culture conditions for 48 h. 3D collagen matrix was prepared by mixing 120 μL of type I collagen solution (A10483-01, Life technologies, Grand Island, NY, USA) with 12 μL of type I collagen FITC-conjugated solution (C4361, Sigma-Aldrich, St. Louis, MO, USA). After incubation, 12 μL of OECM-1 spheroids was incorporated into the collagen mixture. This collagen matrix/spheroids suspension was placed in 24-well plates containing 12 mm coverslips with 150 μL of RPMI medium supplemented with 10% FBS and 1% penicillin–streptomycin solution. To promote matrix polymerization, matrix–spheroid mixtures were incubated for 2 h at 37 °C under a 5.0% CO_2_ atmosphere. In the last 15 min of this incubation, culture medium was replaced with 300 μL of a 1:10,000 Hoechst dilution in supplemented RPMI. After nuclei staining, a 60 μM TRITC-conjugated AsNP treatment was performed for 30 min, and samples were visualized in a Zeiss LSM800 confocal microscope (Carl Zeiss Microscopy, White Plains, NY, USA) at the facility of the Universidad Católica del Norte. The light source was an argon/krypton laser (75 mW), and several optical sections (0.1 mm) per field were captured. Images were acquired as 16-bit, avoiding signal saturation, pinhole adjusted to 1 Airy unit.

### 4.10. Statistical Analysis

Statistical analysis and graphs were performed using GraphPad Prism (RRID:SCR_002798) version 9.0 (GraphPad Software, San Diego, CA, USA). To evaluate the normal distribution and homoscedasticity among our variables, Shapiro–Wilk and Bartlett tests were respectively performed. Parametric analysis was performed by Student’s *t*-test, and Mann–Whitney was considered as a non-parametric alternative. For all cases, significance level was established at α = 0.05, and defining 0.01 ≤ *p* < 0.05 as statistically significant (*), 0.001 ≤ *p* < 0.01 as highly significant (**), and *p* < 0.001 as whole-level significance (***).

## 5. Conclusions

**Figure 7:**
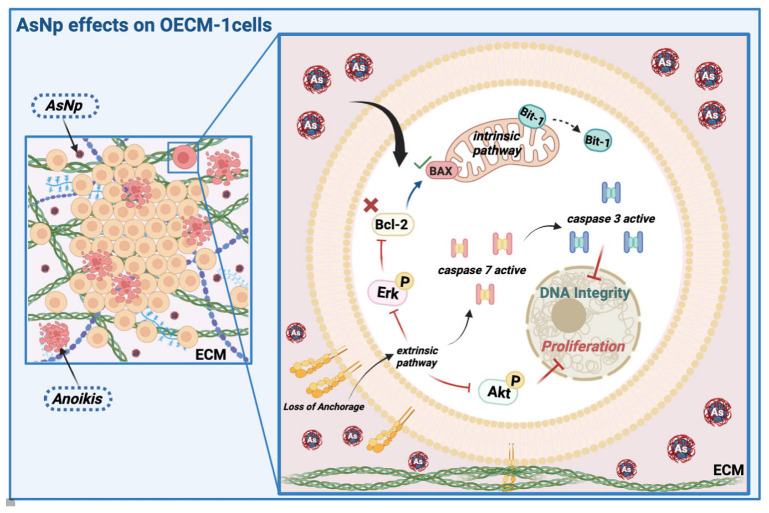
AsNP effects on OECM-1 cells. Arsenic nanoparticles (AsNPs) exert diverse effects in the OSCC cell line OECM-1. After AsNP treatment, an important increase in cell detachment is observed, this triggers a type of apoptosis called *anoikis* (red cells), which can be proven by the increases in Bit-1 relative protein levels, caspases 3/7 activity, Akt/Erk phosphorylation inhibition, and its interaction with ECM components like collagen I. In addition, AsNPs can also be internalized by OECM-1 cells, activating the intrinsic pathway of apoptosis through alterations in the Bcl2/Bax relative protein ratio. Created with BioRender.com (https://app.biorender.com/user/signin; accessed on 13 April 2024).

This study established that AsNPs decrease the viability of the OSCC cell line OECM-1 via apoptosis induction. The extensive cell detachment observed after AsNP treatment suggests that apoptosis is triggered via *anoikis* induction, which was corroborated by the increase in Bit-1 protein levels, complemented by the observed increase in caspases 3/7 activities, and the inhibition of Akt and Erk phosphorylation, which are all key points in the activation of the extrinsic pathway of apoptosis. Aside from these extracellular effects, the intracellular presence of TRITC-conjugated AsNPs was observed by using 3D spheroids. This finding allows to explain that AsNPs can be internalized by OECM-1 cells and lead to the activation of the intrinsic apoptotic pathway through changes in the Bcl2/Bax protein ratio. This confirms that AsNPs reduce cell proliferation by a two-pronged mechanism that targets apoptosis by affecting both *anoikis*-specific and canonical pathways. These changes in cell viability after AsNP treatment (summarized in Figure 7) were not observed in the non-tumoral cell line GES-1, highlighting the potential selectivity of these nanoparticles against cancer cells. These results, although preliminary, describe promising properties for AsNPs as a potential anticancer agent. Future experiments should establish in preclinical and clinical models if these compounds could be useful as an alternative therapeutic approach for oral and skin cancers.

## Data Availability

Data is contained within the article and Supplementary Materials.

## Supplementary Materials

The following supporting information can be downloaded at: https://www.mdpi.com/article/10.3390/ijms25126723/s1.
