# Supplementary material for: Arsenic Nanoparticles Trigger Apoptosis via Anoikis Induction in OECM-1 Cells
Source: Int J Mol Sci. 2024 Jun 18;25(12):6723. doi: 10.3390/ijms25126723 (PMC11204275; doi:10.3390/ijms25126723)
Supplement: Supplementary file 1 [file ijms-25-06723-s001.zip › ijms-2992373-supplementary.pdf]

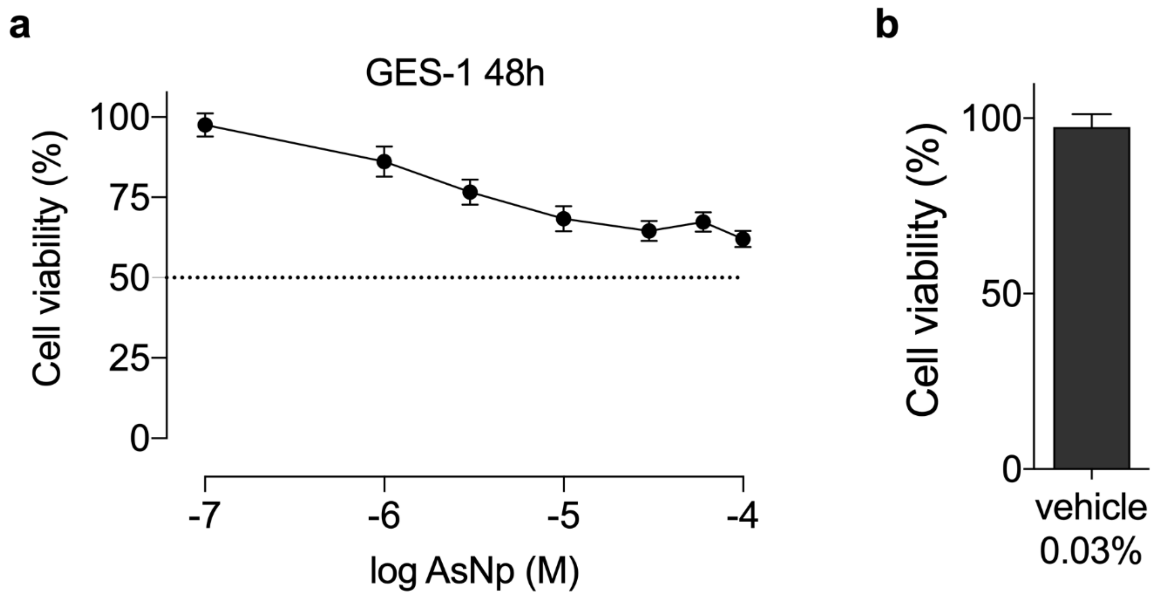

Supplementary figure S1. Viability of GES-1 cells incubated with AsNp. **(a)** Cell viability was measured with Rezasurin© assay. Cells were incubated with crescent concentrations of nanoparticles (1-100 µM and (**b**) included 0.03% vehicle (chitosan) for 48 h. Data are a mean of 4 independent experiments.

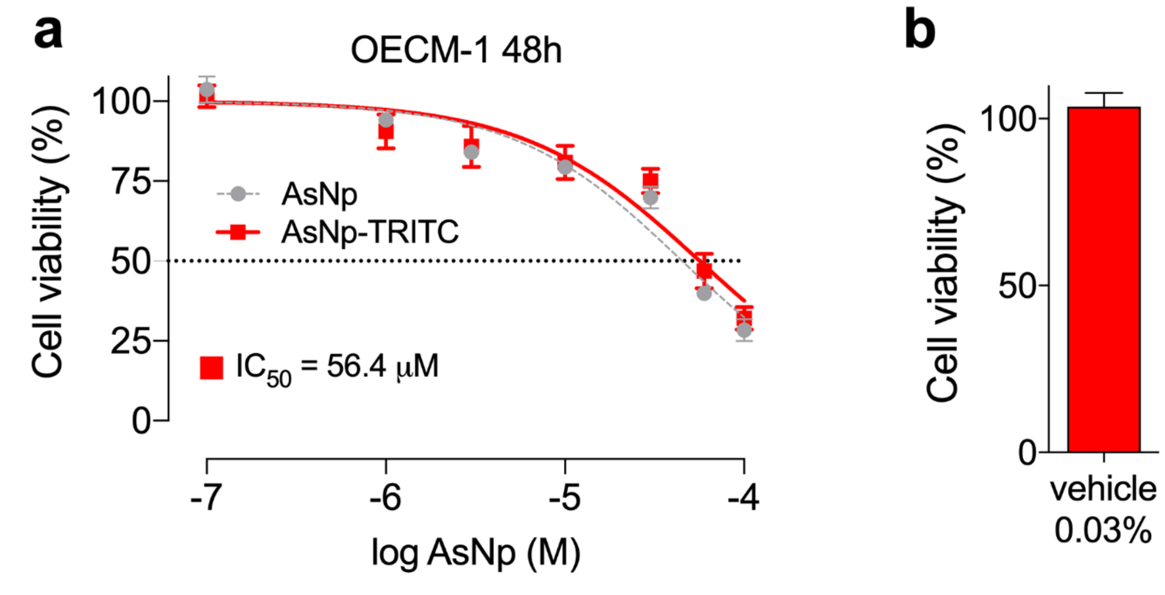

Supplementary figure S2. Effect of TRITC conjugation on AsNp activity against OECM-1 cell proliferation. (a) Cell viability was measured with Rezasurin© assay. Cells were incubated with crescent concentrations of nanoparticles (1-100 µM and (b) included 0.03% vehicle (chitosan) for 48 h. AsNp were incubated alone (gray symbols) or conjugated with TRICT (red symbols and bar). Data are a mean of 4 independent experiments.

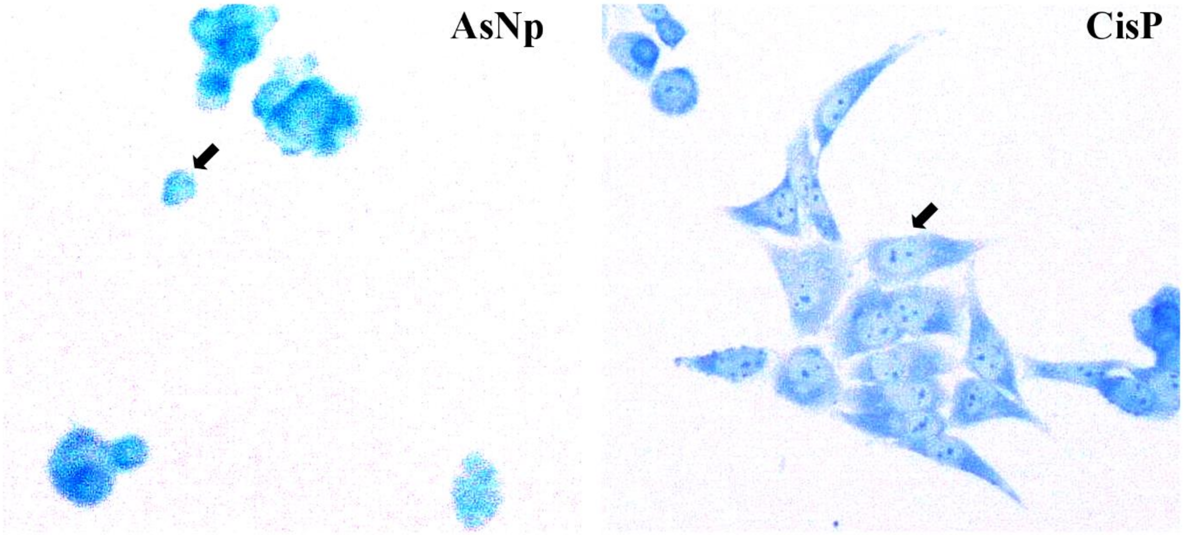

Supplementary figure S3. Morphological differences in OECM-1 cells incubated with AsNp. OECM-1 cells incubated with 60 µM of AsNp (**left**), or 100 **µ**M of CisP (**right**), for 48 h, and stained with 0.2% of methylene blue solution. Arrow indicates the morphological change in these cells.

Table S1. Primer sequences and characteristics for each analyzed gene.

**Gene**	**NCBI Reference Sequence**	**Primer Sequence**	**Amplicon size (bp)**	**Amplicon $T_{melting}$ (°C)**
PTEN	NM_000314.8	Fw: 5′-GCT GGA AAG GGA CGA ACT GGT GTA A-3′	153	77.5 ± 0.5
		Rv: 5′-ATA CAC ATA GCG CCT CTG ACT GGG A-3′		
p53	NM_001276761.3	Fw: 5′-CCC ATC CTC ACC ATC ATC ACA CTG G-3′	222	84.5 ± 0.5
		Rv: 5′-CAG TGG TTT CTT CTT TGG CTG GGG A-3′		
B2M	NM_004048.4	Fw: 5′-AAG TGG GAT CGA GAC ATG TAA GCA-3′	70	77.0 ± 0.5
		Rv: 5′-GGA ATT CAT CCA ATC CAA ATG CGG C-3′		

Table S2. List of antibodies used for immunoblotting.

Antibody	Dilution	Reference
Bcl2	1 : 1,000	Santa Cruz Biotechnology Cat# sc-7382, RRID:AB_626736
Bax	1 : 1,000	Santa Cruz Biotechnology Cat# sc-23959, RRID:AB_626728
BID	1 : 1,000	Santa Cruz Biotechnology Cat# sc-373939, RRID:AB_10917226
AKT (1/2/3)	1 : 1,000	Santa Cruz Biotechnology Cat# sc-81434, RRID:AB_1118808
pAKT (1/2/3)	1 : 1,000	Santa Cruz Biotechnology Cat# sc-81433, RRID:AB_1125472
Erk (1/2)	1 : 1,000	Santa Cruz Biotechnology Cat# sc-514302, RRID:AB_2571739
pErk (1/2)	1 : 1,000	Santa Cruz Biotechnology Cat# sc-81492, RRID:AB_1125801
Bit-1	1 : 10,000	Abcam Cat# ab109291, RRID:AB_10890820
Cleaved caspase-3	1 : 1,000	Cell Signaling Technology Cat# 9661 (also NYUIHC-314, 9661S, 9661L), RRID:AB_2341188
Actin	1 : 4,000	Santa Cruz Biotechnology Cat# sc-8432, RRID:AB_626630
HRP-conjugated anti-rabbit IgG	1 : 5,000	Jackson ImmunoResearch Labs Cat# 711-036-152, RRID:AB_2340590
HRP-conjugated anti-mouse IgG	1 : 5,000	Rockland Cat# 610-1319, RRID:AB_219659
